# What’s New in Intravenous Anaesthesia? New Hypnotics, New Models and New Applications

**DOI:** 10.3390/jcm11123493

**Published:** 2022-06-17

**Authors:** Remco Vellinga, Beatrijs I. Valk, Anthony R. Absalom, Michel M. R. F. Struys, Clemens R. M. Barends

**Affiliations:** 1 Department of Anesthesiology, University Medical Center Groningen, University of Groningen, 9713 GZ Groningen, The Netherlands; b.i.valk@umcg.nl (B.I.V.); a.r.absalom@umcg.nl (A.R.A.); m.m.r.f.struys@umcg.nl (M.M.R.F.S.); c.r.m.barends@umcg.nl (C.R.M.B.); 2 Department of Anesthesiology, University Medical Center Göttingen, 37075 Göttingen, Germany; 3 Department of Basic and Applied Medical Sciences, Ghent University, 9041 Ghent, Belgium

**Keywords:** pharmacokinetics, target-controlled infusion, pharmacology, hypnotics, anaesthetic drugs, anaesthesia

## Abstract

New anaesthetic drugs and new methods to administer anaesthetic drugs are continually becoming available, and the development of new PK-PD models furthers the possibilities of using arget controlled infusion (TCI) for anaesthesia. Additionally, new applications of existing anaesthetic drugs are being investigated. This review describes the current situation of anaesthetic drug development and methods of administration, and what can be expected in the near future.

## 1. Introduction

As in any other area in the medical field, anaesthesia is ever growing and evolving. New drugs are being added to the repertoire and new applications of already widely-used anaesthetic drugs are being discovered. Additionally, the dosing regimens and methods of administration of hypnotic agents are constantly being evaluated and updated using the most recent pharmacological insights, cumulating in advanced pharmacokinetic-pharmacodynamic models that can contribute to the future of personalized medicine.

In this review, we present the current situation of the newest developments in intravenous hypnotic agents. 

## 2. New Hypnotic Agents

**Figure 1:**
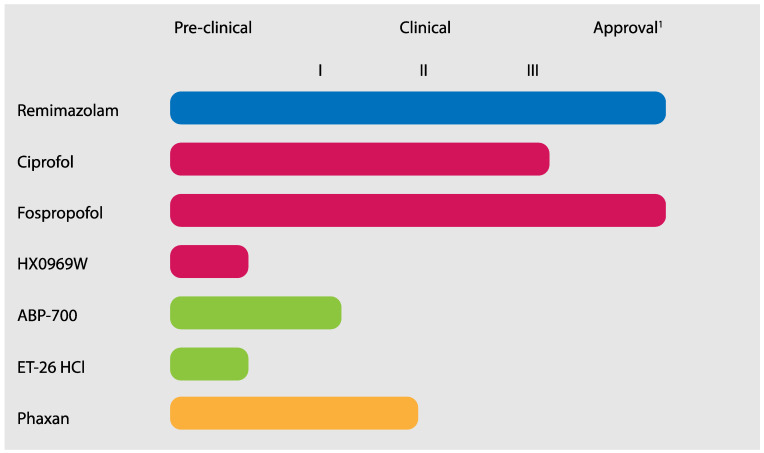
Overview of novel hypnotics and their current status in drug development. ^1^ By the Food and Drug Administration. Blue: Benzodiazepine derivative. Red: Propofol derivatives. Green: Etomidate derivatives. Orange: Steroid.

One area in which the field of anaesthesiology constantly tries to improve itself is the development of new drugs that can be used to induce general anaesthesia and/or sedation. The general aim is to ultimately create a hypnotic that approaches the ideal intravenous (i.v.) anaesthetic agent that has the following characteristics: It should have a rapid onset of action and an equally rapid, organ independent, clearance, so that its effect can be tightly controlled. Furthermore, the ideal i.v. anaesthetic agent does not affect circulatory and respiratory systems in a negative way and has a minimal amount of side effects. Ideally, it should also have additional desirable properties, such as anti-emetic or analgesic effects [1]. None of the i.v. anaesthetic agents currently meet all of these requirements. Currently, several novel hypnotic compounds are in development (Figure 1). This review describes the currently available published literature on new hypnotic agents. At the moment of writing, several of the mentioned new hypnotics are being investigated in clinical trials. Trial registration can be found at clinicaltrials.gov and in the clinical trial databases of individual countries. For a complete overview about pharmacokinetic and pharmacodynamic parameters of established hypnotic agents and new hypnotic agents in the clinical phase of drug development, see Table 1. It has to be considered, however, that, especially for the new hypnotics, much of the data is preliminary and/or based on a small sample size. These data, therefore, have to be interpreted with caution.

### 2.1. Mechanism of Hypnotic Agents

In order to design and develop new hypnotic agents, it is important to have knowledge of the mechanism of action of current anaesthetic agents, and thus of their desired clinical effects. The molecular mechanisms of the clinical drug effect of anaesthetic agents have been an enigma to researchers for a long time, not in the least because of the large chemical diversity among current anaesthetic drugs. Only during the last three decades [12] has the γ-aminobutyric acid type A (GABA_A_) receptor been shown to be the most likely molecular target for most intravenous general anaesthetic agents [13,14,15]: it is the main molecular target for the hypnotic agents propofol and etomidate and the barbiturates and the benzodiazepines.

The GABA_A_ receptor is a transmitter-gated ion channel located on synapses throughout the central nervous system (CNS). It is composed of five transmembrane-crossing subunits which surround a central ion channel pore. The GABA_A_ receptor produces its effect when it is activated by the matching neurotransmitter, GABA. Upon activation, the receptor undergoes a conformational change, allowing the centre ion channel pore to open. This permits extracellular chloride ions to pass into the intracellular space, resulting in hyperpolarization of the neuron, thus inhibiting the activity of that particular cell [16]. Anaesthetic agents bind to distinct binding sites on the GABA_A_ receptor. Binding causes a conformational change and the influx of chloride ions is increased. This is called positive allosteric modulation. The mechanism behind this enhancement and its extent depend on the anaesthetic and its binding site on the GABA_A_ receptor.

Not all hypnotic and sedative agents act at the GABA_A_ receptor. Ketamine has no affinity for the GABA_A_ receptor but instead blocks the stimulation of the CNS by acting as a noncompetitive antagonist of the CNS stimulating *N*-methyl-D-aspartate (NMDA) receptor [4]. Dexmedetomidine is also not a GABA_A_ receptor agonist. It is an α_2_ receptor agonist and exerts its action by stimulating the locus coeruleus and by inhibiting the sympathetic nervous system.

### 2.2. New Hypnotic Agents Approved for Clinical Use

#### 2.2.1. Remimazolam

Two types of salt forms of remimazolam have been registered, remimazolam besylate (PAION UK Ltd., London, UK) and one using a slightly different salt form, remimazolam tosylate (Jiangsu Hengrui Pharmaceutical Co., Ltd., China) [17]. Remimazolam is a new ultra-short-acting benzodiazepine [7]. It exerts its anaesthetic effects by binding to the benzodiazepine binding site at the GABA_A_ receptor. The compound is rapidly metabolized to an inactive metabolite by CES 1 tissue esterases, which make it a new ultra-short-acting drug. Remimazolam has recently been approved for procedural sedation in the USA, China and Europe. It is approved for general anaesthesia in Japan and South Korea [6]. Regulatory assessment for general anaesthesia in other countries will follow. 

There are several studies that have shown that remimazolam is well tolerated and is effective for procedural sedation [7,18,19,20]. Remimazolam causes an increase in heart rate and a decrease in blood pressure at the start of the infusion [7]. Onset of sedation is rapid compared to midazolam [21], even though it is less potent [22]. Single boluses combined with top-up doses provide sufficient sedation levels for a colonoscopy without the need for mechanical ventilation [23]. Rex and colleagues [24] showed that less fentanyl was needed during colonoscopy and that patients recovered faster and were ready for discharge earlier than with midazolam. They furthermore reported a lower incidence of hypotension, but a similar frequency of hypoxia compared to midazolam. Studies are being conducted to gain more information about safety and dosing in children. Several studies [20,24,25], have shown that remimazolam, in contrast to propofol, does not induce pain during infusion, which would be beneficial in paediatric practice. One of the more important favourable characteristics of remimazolam is that, unlike other current anaesthetic agents, its effects can be antagonized with flumazenil.

Remimazolam is non-inferior to propofol for general anaesthesia in ASA II and III patients [26,27] with respect to safety and efficacy. Furthermore, it appears to induce loss of consciousness more slowly than propofol [6]. Detailed information about the safety and efficacy of remimazolam compared to propofol in ASA III and IV patients should emerge soon from the results of a recently completed large multi-centre study (reference number NCT03661489).

There are two important caveats associated with remimazolam use. Remimazolam precipitates when mixed with Ringers’ acetate or Ringers’ lactate solutions [28]. Therefore, co administration with other intravenous fluids is needed. Additionally, there is emerging evidence that remimazolam’s use is associated with the occurrence of delirium, despite the potential benefit of the fast offset of action [29]. These topics still need further research.

Currently, several studies concerning remimazolam are being conducted. A trial about the interaction between remifentanil and remimazolam was recently completed (NCT0467047), and a multicentre study about the comparison between midazolam and remimazolam in ventilated patients is recruiting. For a complete overview about all studies currently conducted see clinicaltrials.gov.

#### 2.2.2. 2,6-Disubstituted Phenol Derivatives

Propofol is currently the best-known 2,6-disubstituted phenol derivative, and in many aspects, it is considered to be the gold standard in general anaesthesia and procedural sedation. It acts on the GABA_A_ receptor through positive allosteric modulation (see above). Propofol has many of the properties of the ideal anaesthetic agent: it has a fast onset of effect (within 30s), a short duration of action leading to a rapid recovery free from hangover effects, and research indicates predictable pharmacokinetics. Additionally, it has amnestic [30] and anxiolytic [31] effects as well as antiemetic potential [32]. It has several significant side-effects, however, such as marked cardiovascular and respiratory depression and pain on injection [33]. Furthermore, it can cause metabolic derangements such as hyperlipidaemia, attributable to the lipid formulation of propofol [34].

#### 2.2.3. Propofol Analogues

Efforts have been made to reduce propofol’s unwanted side effects, and currently two molecules are in development that have a similar chemical structure to propofol for use in general anaesthesia or sedation: ciprofol and fospropofol.

##### Ciprofol

Ciprofol (2-(1-Cyclopropylethyl)-6-isopropylphenol), or HSK3486 (Haisco Pharmaceutical Group Co., Ltd., Chengdu, China), was initially developed for use in anaesthesia, for procedural sedation during invasive endoscopy and for adult intensive care sedation. It was initially developed as one in a series of 2,6-disubstituted alkylphenols. The aim of the development of this series was to decrease the lipophilicity of propofol and to enhance the binding on the GABA_A_-receptor [35]. In vitro and in vivo data showed that ciprofol also causes a rapid onset of hypnotic effect, as well as a rapid recovery. Phase I trials of ciprofol conducted in Australia and China confirmed these findings in humans, reporting short median terminal half-lives and a short time to peak hypnotic effect (within 2 min) as well as a quick recovery (5–14 min) [36,37]. The maximum concentration (C_max_), the time to the maximum concentration, the terminal elimination half-life (*t*_1/2_) and the mean residence time (MRT) of ciprofol appeared similar to those of propofol, but clearance (*CL*), volume of distribution(*V_d_*) and volume of distribution at steady state (*V_SS_*) were all significantly larger in propofol [36]. For ciprofol, the occurrence of “abnormal limb movements” has also been described during induction with higher dosages [8], similar to involuntary muscle movements observed with the established hypnotics etomidate and propofol, and the experimental hypnotics ABP-700 [9] and AZD3043 [38]. Subsequent phase II and III studies in patients undergoing gastrointestinal endoscopy and fiberoptic bronchoscopy described non-inferiority in the success rate of the procedure of bolus doses of ciprofol compared to bolus doses of propofol. The main advantage of ciprofol in these studies was a lower incidence of pain on injection in patients who received ciprofol [39,40]. A phase III trial is planned which will compare ciprofol to propofol for intensive care sedation [41]. Currently, ciprofol is registered in the US only for the induction of general anaesthesia [42]. In China, phase III studies for the maintenance of general anaesthesia are being conducted and ciprofol is approved for use in sedation for colonoscopy and for the induction of general anaesthesia.

##### Fospropofol

Fospropofol (LUSEDRA^®^, Eisai, Inc. Woodcliff Lake, NJ, USA) was developed as a prodrug of propofol in an effort to reduce the injection pain commonly seen with propofol administration. As a prodrug, fospropofol is an inactive compound, and it is metabolized by alkaline phosphatases into the active compound propofol and formaldehyde. Fospropofol does not need to be formulated in a lipid solution, which should reduce the incidence of pain on injection and the risk of metabolic side effects, and it provides a more stable formulation [43]. Clinical trials in patients undergoing colonoscopy or flexible bronchoscopy showed that fospropofol is well tolerated, although a significant number of patients reported burning perineal pain after receiving fospropofol. In a phase II-trial in 240 patients by Liu et al. this disconcerting and ill-understood side-effect occurred in 95% of the subjects [44]. These findings contributed to the limited use of fospropofol in daily clinical practice. Currently, fospropofol is approved in the United States for use in sedation in adults undergoing diagnostic or therapeutic procedures, but not for use as a general anaesthetic.

##### HX0969W

HX0969W is another propofol prodrug that has been in preclinical development. It is metabolized to propofol and gamma-hydroxybutyric acid. In vitro and in vivo data showed that HX0969W has a slower onset of action but a longer duration of loss-of-righting-reflexes in rats compared to propofol [45]. No human trials of HX0969W have been published so far.

#### 2.2.4. Etomidate Analogues

Etomidate is also a GABA_A_ receptor agonist introduced into clinical practice in 1972 for use in both general anaesthesia and sedation. Etomidate is particularly well known for its haemodynamic and respiratory stability [46]. In 1982, however, an increased mortality was reported in ICU patients who received etomidate by continuous infusion [47]. This was shown to be caused by etomidate-induced inhibition of the 11β-hydroxylase enzyme causing adrenocortical suppression [48]. Since then, the use of etomidate has been limited to the induction of general anaesthesia in select patient groups, in whom the proposed haemodynamic stability after etomidate induction outweighs the risk of adrenal suppression [3]. Since the late 2000s, analogues of etomidate have been in development with the aim to eliminate the adrenocortical suppressive action while retaining its haemodynamically stable profile.

##### ABP-700

ABP-700 (cyclopropyl-methoxycarbonyl-metomidate) is one of several drugs in a series of etomidate analogues that have been developed by Husain et al. with the goal of reducing adrenocortical suppression [49]. From this series, ABP-700 showed the most favourable pharmacological properties both in vitro and in vivo in rodents and Beagle dogs, without the occurrence of adrenocortical suppression, and the drug was subsequently advanced to phase I studies [50]. These volunteer studies showed that ABP 700 has an extremely rapid on- and offset of hypnotic action, with onset of hypnotic effect occurring within 30 s after a bolus dose, and recovery occurring mostly within 5 min. ABP-700 also appeared to have a stable haemodynamic and respiratory profile, and adrenocortical suppression was not detected [9,51]. An important side effect, however, was the occurrence of involuntary muscle movements. These movements ranged from the slight twitching of a single limb to full body myoclonic movements. This led to a interruption in the further development of the drug. A recirculatory model of ABP-700 demonstrated that this phenomenon is likely attributable to a disequilibrium in inhibitory and excitatory neuronal circuits in the brain during the induction of anaesthesia [52].

##### Preclinical Developments

Another etomidate-analogue, currently in the preclinical development stage, is ET-26 Hydrochloride. In vivo, ET-26HCl provided a stable haemodynamic profile with hypnotic properties similar to etomidate, and it caused significantly less adrenocortical suppression [53,54]. No clinical trials in humans have been reported.

#### 2.2.5. Neurosteroids

Neurosteroids also work as positive allosteric modulators on the GABA_A_-receptor. Endogenous neurosteroids, for example, are thought to act as an analgesic during pregnancy [55]. Alfaxalone (3α-hydroxy-5α-pregnane-11,20-dione, or Althesin^®^)) is an exogenous neurosteroid and analogue of progesterone that was developed as a general anaesthetic [56]. From 1972 to 1984, it was used in daily clinical practice, and it displayed a rapid onset and short duration of effect, a large therapeutic index, and minimal cardiovascular and respiratory depression [57]. It was withdrawn, however, because of hypersensitivity reactions to the excipient CremaphorEL^®^. Since then, alfaxalone is mainly used in veterinary practice. Because alfaxalone is highly lipid soluble, it is difficult to formulate it in a compound that is suitable for intravenous administration in humans [57].

In order to improve the formulation while retaining the favourable clinical profile of alfaxalone in humans, a new formulation of alfaxalone in an aqueous solution in 13% 7-sulfobutyl ether β cyclodextrin (SBECD/betadex) was produced: phaxan. In preclinical and clinical studies, phaxan demonstrated a fast-onset and short duration of clinical effect [11,58]. A pharmacokinetic-pharmacodynamic study of phaxan showed a high plasma clearance, and, although it demonstrated a relatively long half-life, it had a fast on- and offset of anaesthetic action [10].

## 3. New PK-PD Models for Existing Anaesthetic Drugs for TCI Application

The accuracy of intravenous drug administration can be improved by the use of target controlled infusion (TCI) systems [59]. TCI systems use computer driven infusion pumps with pharmacological models incorporated into the software to reach and maintain a user-defined plasma or effect site concentrations. In doing so, TCI systems calculate the bolus and infusion rate to achieve a specific plasma or effect-site target concentration considering the simultaneous pharmacokinetic processes of distribution and elimination clearance from the body. TCI systems facilitate the administration of reasonably stable concentrations and facilitates rational titration in drug plasma concentrations during procedures. For several anaesthetic drugs, pharmacological models have been developed that can be used in the aforementioned infusion pumps and for some drugs more than one model exists.

The performance of these models (i.e., their precision, variability and bias) can be compared using the Varvel criteria [60]. This is a multistep process in which the bias (MdPE), precision (MdAPE), and the variability (Wobble) of a model are determined by comparing drug concentration measurements with the concentrations predicted by the model. A MdPE < 20% and a MdAPE < 40% have been suggested to indicate clinically acceptable performance [61,62]. Knowing the performance of a model aids in selecting the optimal model for a specific patient. A detailed description of the demographic ranges of commercially available models can be found in a recent publication by Vandemoortele et al. [63].

Marsh [64] and Schnider [65,66] both published a TCI model for adults and the Kataria [67] and Paedfusor [68] propofol models have both been developed for use in children. Recently, the first general purpose model, the Eleveld-model, for propofol became available and was validated in clinical practice [69]. The Eleveld PK-PD model [70] is unique in that it can be used for target-controlled infusions in children, adults, older subjects and the obese [69,71].

Using a general purpose model designed for a broad population range increases the match between a model and a clinical situation [72], thus reducing the risks associated with extrapolation, incorrect usage and unfamiliarity with the TCI-model.

A more in-depth description about the performance of the models is shown in Table 2. The Eleveld model has a low bias (MdPE < 20%) for children, adults and the obese, but the bias in the elderly (27%) is larger. Some of the other commercially available models have a lower average pharmacokinetic MdAPE compared to the Eleveld model. This difference is not, however, statistically significant at an alpha- level of *p* < 0.01.

The Schnider model has a lower MdPE for adults and the elderly, and it is unsuitable for use in children. For the paediatric patient, the Paedfusor model is best selected, because it has a lower bias and lower precision, although these performance measures do not differ significantly from the Eleveld model.

For obese patients, several considerations need to be considered when selecting a model. The Schnider model cannot be used in female patients with a BMI over 37 kg m^−2^ and male patients with a BMI of more than 42 kg m^−2^. The Marsh model has a higher PK bias and better PK precision than the Eleveld model. The latter model, however, has a very good pharmacodynamic performance profile with an MdPE of less than 2% and an MdAPE of less than 10%.

There are two commercially available models for dexmedetomidine, the Dyck-model [73] and the Hannivoort-Colin model [74,75,76]. The Hannivoort-Colin model has a PD component for four different pharmacodynamic endpoints: BIS, the Modified Observer’s Assessment of Alertness/Sedation (MOAA/S) score, heartrate, and mean arterial pressure. It can be safely used for plasma targets up to 2 ng mL^−1^ because it assumes linear pharmacokinetics [77] but at higher plasma concentrations, the kinetics become non-linear, and may result in higher plasma concentrations than expected.

For remimazolam there is currently no commercial TCI model available. Zhou and colleagues [78] have published a three-compartment PK-PD model with BIS as an endpoint. The model was extended with MOAA/S [79] scores, because MOAA/S scores are more accepted as pharmacodynamic endpoints for sedation. In this model, age has no effect on pharmacokinetics, but a pharmacodynamic effect on age was found. As age increases, the duration of sedation following a single bolus increases. Zhou and colleagues describe this as clinically irrelevant and suggest that a dose reduction for the elderly is not needed. One of the limitations of this model is that the predicted MOAA/S scores were less well matched compared to the observed MOAA/S scores. The model was able to predict appropriate MOAA/S scores for bronchoscopy, but overestimated the effect for colonoscopy. These limitations are not useful for a TCI model and need to be improved. 

## 4. New Drug Applications

For trypanophobic patients, or those lacking the cognitive abilities to cope with the discomfort of an injection or venepuncture, extravascular administration routes can provide comfortable and tolerable ways of inducing sedation and hypnosis. Although oral administration is arguably the most obvious route of extravascular administration, the limited oral bioavailability, coupled with unpredictable C_max_ and T_max_ limit the clinical usability of this route.

Intranasal administration of two known hypnotic drugs, dexmedetomidine and midazolam, has, however, proven to be both predictable and clinically useful in selected patient groups.

The nasal mucosa absorb both drugs well and for children and younger adults the use of both dexmedetomidine [80,81,82] and midazolam for sedation and premedication has been studied. Refs. [83,84] and the pharmacokinetics and pharmacodynamics after intranasal administration of dexmedetomidine and midazolam have been described. Both drugs can be used in this way to provide anxiolysis during premedication or for procedural sedation.

Most studies of intranasal administration of dexmedetomidine use the intravenous formulation of 100 µg mL^−1^. The bioavailability of dexmedetomidine after IN administration is 40.6% (95%-CI 34.7–54.4%) [85], and the T_max_ has been found to be 75–78 min with the onset of clinical sedation at 20-30 min after administration [80,85,86]. Although elderly patients are considered to be more prone to sedative effects of most hypnotic drugs, a comparison of the results of two studies (one in younger adults and one in geriatric subjects) contradicts this concept. In the study of Barends et al. where elderly patients received intranasally administered dexmedetomidine, only 45% of the subjects attained a MOAA/S-score of 3 or lower after receiving dosages of 1.0 µg kg^−1^ and 1.5 µg kg^−1,^ while in the study of Yuen et al., involving younger adults, 75% and 92% of the subjects reached a MOAA/S score of ≤3 after these doses [82].

While dexmedetomidine is known for its complex haemodynamic consequences, the effects on blood pressure and heart rate of intranasal dexmedetomidine administration in non-geriatric adults are relatively benign [80,81,82]. In geriatric subjects treated with dosages used for younger adults (1.0–2.0 µg kg^−1^), however, intranasal dexmedetomidine resulted in unacceptable levels of hypotension, while sedation levels remained relatively light. The use of intranasal dexmedetomidine in elderly patients was discouraged by the authors because of these findings [86]. Intranasally administered dexmedetomidine may play a useful role as a sedative hypnotic agent in children and younger, healthy adults, but care should be taken when its use is planned in elderly patients.

The intranasal administration of midazolam is not new. The intravenous formulation can be dripped or sprayed into the nose to achieve clinical sedation. This use, however, is associated with nasal irritation and reduced predictability because of the large volume of midazolam that needs to be administered and the pH of intravenous midazolam, which renders it hydrophilic rather than lipophilic. Newer midazolam formulations designed specifically for intranasal administration have recently been developed and the pharmacokinetics of intranasal midazolam have been studied for a number of formulations and dosages [87,88,89]. For the intranasal use of midazolam in adults, most information comes from neurological literature where seizure control is the primary indication [84], but the use of intranasal midazolam in surgical and dental adult patients has also been described [83,84,90].

The formulations used in these studies are highly concentrated (up to 55.6 mg mL^−1^) to allow a small volume to be atomized or to be delivered through unit-dose spray of 70–90 µL to the nasal mucosa. Furthermore, the pH of these formulations has been adapted to make the drug less irritating to the nose and to improve absorption. Most intranasal midazolam compounds are water-based formulations with additives such as propylenegycol and an alcohol as a preservative [84]. Recently a chitosan-based formulation was tested as well. This product, however, caused nasal irritation and other symptoms of discomfort in 83–89% of subjects [89]. This is arguably of lesser concern within the investigated indication of emergency rescue medications for epileptic patients. 

Intranasal midazolam has been shown to have a fast and reliable onset time in children and younger adults. It is absorbed rapidly, consistently and extensively [84]. T_max_ after IN administration lies between 14 and 20 min [84,87,89]. In non-geriatric adults the bioavailability of the water-based compound is approximately 60% [88].

While dexmedetomidine is often preferred over midazolam for procedural sedation because of the supposed benefit of less respiratory depression, in the published studies on intranasal midazolam respiratory depression was rare [89,90,91].

## 5. Conclusions/Summary

In the past decade, many developments have taken place in the field of intravenous anaesthesia. Remimazolam is a promising new drug that may become more widely used in daily clinical practice, but other compounds, such as ciprofol and phaxan, have shown promising results as well. For improved intravenous drug administration, new PK-PD models of intravenous anaesthetics, the latest generation [92] of TCI systems, are able to handle the higher complexity of incorporating patient characteristics (weight, height, age, sex, and additional biomarkers), and this may improve the accuracy when administering existing hypnotic drugs.

Dexmedetomidine and midazolam have both been shown to be effective and safe for the procedural sedation of children and healthy adults after intranasal administration. The development of formulations specific for this administration route can improve the efficacy of those drugs. Such formulations have been developed for midazolam, while for dexmedetomidine there is, to our knowledge no special intranasal formulation.

## Figures and Tables

**Table 1:** Pharmacokinetic and pharmacodynamic parameters of established hypnotic agents and new hypnotic agents in the clinical phase of drug development. PONV = postoperative nausea and vomiting. Data after an established induction bolus dose. Values are represented as ranges or mean ± SD. o is no effect, -- strong negative, - less negative, ++ strong positive, + positive, ? is unknown.

	Mechanism of Action	Elimination Half-Life (min)	Clearance (mLkg^−1^min^−1^)	Vdss (L kg^−1^)	Time to Onset (s)	Effect on							Reversal with Flumazenil	Significant Side Effects
						Heart Rate	Blood Pressure	Respiration	Amnesia	PONV	Analgesia	Pain on Injection		
Propofol [2]	GABA_A_ receptor	116–834	25.4–32.6	2.27–11	<30	o	--	--	++	--	o	++	o	Propofol infusion syndrome
Etomidate [3]	GABA_A_ receptor	174–330	9.9–25.0	0.15–4.7	<30	o	o	o		+	o	+	o	Adrenal suppression; Excitation
Ketamine [4]	NMDA receptor	120–240	14.3–35.0	2.28–7.86	<60	++	++	o	+	-	++	o	o	Psychedelic effects, Elevated Intracranial pressure
Midazolam [5]	GABA_A_ receptor	102–156	6.4–11	1.1–1.7	120–180	+	-	+	++	+	o	o	+	
Remimazolam [6,7]	GABA_A_ receptor	70 ± 10	1.15 ± 0.12	35.4 ± 4.2	60–90	+	-	+	++	+	o	o	+	
Ciprofol [8]	GABA_A_ receptor	105.6–125.3	21.7–24.6	3.6–4.3	60–120	-	-	?	?	o	?	o	o	
ABP-700 [3,9]	GABA_A_ receptor	13.1–16.2	27.8	?	<30	+	+	+	?	+	o	o	o	
Phaxan [10,11]	GABA_A_ receptor	?	15.4	0.38	<60	o	-	o	?	o	?	o	o	

**Table 2:** The predictive performance for PK (plasma concentrations) and PD (BIS) of the Eleveld PK-PD model compared to other propofol models. (Reproduced with permission [71], 2020, British Journal of Anaesthesia.)

Arterial Samples	MdPE (%)	MdAPE (%)	ΔMdAPE (%)	*p*-Value	Wobble (%)	ΔWobble (%)	*p*-Value
**Eleveld PK-PD**							
Children	−4.42 (−35.1, 37.6)	16.8 (1.92, 37.6)			7.39 (0.77, 16.0)		
Adults	−14.1 (−43.3, 24.6)	19.5 (4.86, 43.3)			7.89 (2.01, 17.6)		
Elderly	−27.0 (−53.9, 7.75)	29.5 (6.02, 53.9)			7.28 (2.48, 24.7)		
Obese	−14.1 (−40.7, 11.6)	18.3 (4.11, 40.7)			6.80 (1.89, 14.1)		
**Schnider**							
Adults	−2.11 (−35.6, 61.8)	17.5 (6.04, 61.8)	−1.98 (−15.7, 37.2)	0.020	8.81 (4.11, 22.2)	0.92 (−3.54, 5.99)	0.989
Elderly	−5.13 (−44.0, 46.2)	22.2 (5.68, 46.2)	−7.28 (−23.7, 36.2)	0.043	9.46 (1.74, 25.8)	2.18 (−1.65, 10.0)	1.000
Obese	5.75 (−30.8, 56.2)	22.1 (5.02, 56.2)	3.80 (−21.1, 46.0)	0.674	8.08 (1.97, 24.3)	1.28 (−7.09, 12.1)	0.971
**Marsh**							
Adults	−0.03 (−24.8, 53.1)	16.4 (5.82, 53.1)	−3.14 (−25.3, 29.4)	0.051	9.33 (3.33, 19.6)	1.43 (−4.34, 8.01)	0.996
Elderly	3.38 (−45.9, 80.4)	26.2 (7.85, 80.4)	−3.23 (−32.5, 63.2)	0.150	12.0 (4.53, 44.4)	4.70 (−1.47, 19.8)	1.000
Obese	17.0 (−32.7, 71.3)	28.8 (6.48, 71.3)	10.5 (−23.1, 62.7)	0.965	9.46 (3.43, 18.6)	2.66 (−5.20, 6.95)	1.000
**Marsh (Servin-formula)**							
Obese	−10.7 (−46.2, 30.9)	20.0 (2.16, 46.2)	1.69 (−15.7, 22.3)	0.914	7.30 (2.65, 14.0)	0.50 (−6.74, 4.07)	0.957
**Cortinez (Obese)**							
Obese	−7.48 (−47.3, 32.0)	20.0 (4.22, 47.3)	1.70 (−12.9, 23.4)	0.738	7.67 (1.25, 18.8)	0.87 (−3.73, 5.36)	0.983
**Paedfusor**							
Children	−3.18 (−27.1, 38.1)	15.5 (2.57, 38.1)	−1.37 (−13.9, 10.2)	0.262	9.89 (2.06, 25.0)	2.50 (−10.7, 15.9)	0.995
**Kataria**							
Children	26.2 (−12.9, 95.8)	31.1 (7.59, 95.8)	14.3 (−16.6, 70.1)	0.995	12.6 (0.95, 38.1)	5.25 (−5.28, 22.1)	0.999
**BIS**	**MdPE (BIS)**	**MdAPE (BIS)**	**ΔMdAPE (BIS)**	***p*−Value**	**Wobble (BIS)**	**ΔWobble (BIS)**	***p*−Value**
**Eleveld PK-PD**							
Children	1.95 (−21.7, 20.9)	9.10 (3.43, 21.7)			4.35 (1.73, 8.94)		
Adults	0.29 (−15.2, 17.9)	7.88 (1.95, 17.9)			3.60 (1.55, 7.45)		
Elderly	1.80 (−11.9, 13.1)	7.57 (2.69, 13.1)			4.46 (2.18, 8.28)		
Obese	0.74 (−24.4, 31.3)	9.61 (3.17, 31.3)			3.50 (1.76, 6.03)		

## Data Availability

Not applicable.
